# Formation of solid helical filaments at temperatures of superfluid helium as self-organization phenomena in ultracold dusty plasma

**DOI:** 10.1038/s41598-019-40111-w

**Published:** 2019-03-01

**Authors:** Roman E. Boltnev, Mikhail M. Vasiliev, Evgenii A. Kononov, Oleg F. Petrov

**Affiliations:** 1 Joint Institute for High Temperatures, Russian Academy of Sciences, Moscow, 125412 Russia; 2 Branch of Talrose Institute for Energy Problems of Chemical Physics, Russian Academy of Sciences, Chernogolovka, Moscow region, 142432 Russia; 3 Moscow Institute of Physics and Technology, Dolgoprudnyi, Moscow region, 141701 Russia

## Abstract

A multimodal dusty plasma formed in a positive column of the direct current glow discharge at superfluid helium temperatures has been studied for the first time. Formation of a liquid-like dusty plasma structure occurred after injection of polydisperse cerium oxide particles in the glow discharge. The coupling parameter ~10 determined for the dusty plasma structure corresponds very well to its liquid-like type. The cloud of nanoparticles and non-linear waves within the cloud were observed at T < 2 K. Solid helical filaments with length up to 5 mm, diameter up to 22 μm, total charges ~10^6^е, levitating in the gas discharge at the temperature ~2 K and pressure 4 Pa have been observed for the first time. Analysis of the experimental conditions and the filament composition allows us to conclude that the filaments and nanoclusters were formed due to ion sputtering of dielectric material during the experiments.

## Introduction

Self-organization phenomena in the nature are extremely diverse. They can be found in dissipative systems of varying complexity and scale: from physical systems studied in nanoworld and astronomy up to social and economic processes in the human world. The phenomena of self-organization are inherent for open non-equilibrium systems consisting of non-linearly interacting components. Dusty plasma formed by microscopic charged particles levitating in gas discharge plasma is an example of such systems. Intense scattering of light by microscopic particles allows straightforward observation of particle motions and determination of their coordinates and velocities. This is why a dusty plasma can be used as a convenient tool to study various self-organization phenomena, for example, 3-D^1–5^ and 2-D^6^ phase transitions, formation of non-linear waves^7,8^, including rogue waves^9^. Dusty plasma provides unique opportunity to carry out studies within much wider range of neutral gas temperature (temperature changes by at least two orders of magnitude in the case of helium gas) as compared to alternative systems, such as clusters composed of water droplets levitating above hot surfaces^10–13^. Therefore, the properties of dusty plasma and different processes in this plasma can be investigated at different temperatures of neutral gas.

Not many comparative experimental studies of dusty plasmas at 4.2 K and 77 K have been conducted so far^14–19^. The results of these studies have been recently reviewed and analyzed in^20^. Contradictory results on the temperature dependence of the interparticle distance in dusty plasma structures have been discussed in^20^. A formation of superdense ordered dusty plasma structures at the lowest temperatures was expected for many years based on the assumption of the validity of a direct relation between the kinetic energy of microscopic particles and the neutral gas temperature^15,19^. Nevertheless, this assumption has not been confirmed experimentally^20^. Another interesting and still unexplained experimental result is a decrease in the dust particle charge with a decrease in the neutral gas temperature^16–18^. No experiments with plasmas at temperatures below 4.2 K have been reported in the literature up to date. Thus the question regarding the lowest possible temperature in dusty plasma systems still remains open. This temperature is of great importance for experimental studies of properties of ultracold dusty plasmas.

However, experimental studies of ultracold dusty plasmas can generate very promising results because cryogenic gas discharges are not well studied for temperatures below 5 K. The main problem in developing and studying of such plasma systems is not cooling the discharge tube down to a temperature below 4.2 K (liquid helium can be used as a cryo-coolant), but restriction of the power released in the gas discharge and increased neutral gas temperature^21^. There is no reliable information in the literature on the positive charge carriers in cryogenic glow discharge because even at T≈80 K the densities of molecular ions, He_3_^+^ and He_4_^+^, reach 10% и 1%, respectively^22,23^. It is known that the conductivity mechanism of the helium plasma is changing at T < 5 K^24^ and such plasma doesn’t emit light^21^. Consideration has to be given to a possible presence of metastable negative helium ions, He^−^ and He_2_^−^, with the life times 359 μs^25^ and 135 μs^26^, respectively, in the helium plasma at T~1 K. Formation of both helium anions, He^−^ and He_2_^−^, involves metastable He* atoms^27^. The density of He* atoms may reach values ~10^13^ cm^−3^ at the total density of helium atoms ~10^17^ см^−3^ and T≈10 K^28,29^.

In this work, we present new experimental results on dusty plasma in direct current (DC) glow discharge inside a tube cooled with superfluid helium^30^. Cooling the neutral and positive ionic components of plasma down to temperatures ≈1 K allows such plasma to be classified as ultracold plasma^31^. Thus, ultracold and strongly coupled dusty plasma with coupling parameter of ~10 has been observed at T < 2 K for the first time. Moreover, a spheroidal plasma dusty structure formed by CeO_2_ particles in the stratum of positive column of the glow discharge cooled by superfluid helium was observed together with a cloud of weakly charged nanoclusters with sizes less than 100 nm and with separate levitating solid filaments with the length of a few mm’s. Based on the results obtained in the present study it can be concluded that nanoclusters and solid filaments were formed within the discharge volume due to ion sputtering of dielectric material in the discharge tube at T < 2 К.

## Methods

**Figure 1:**
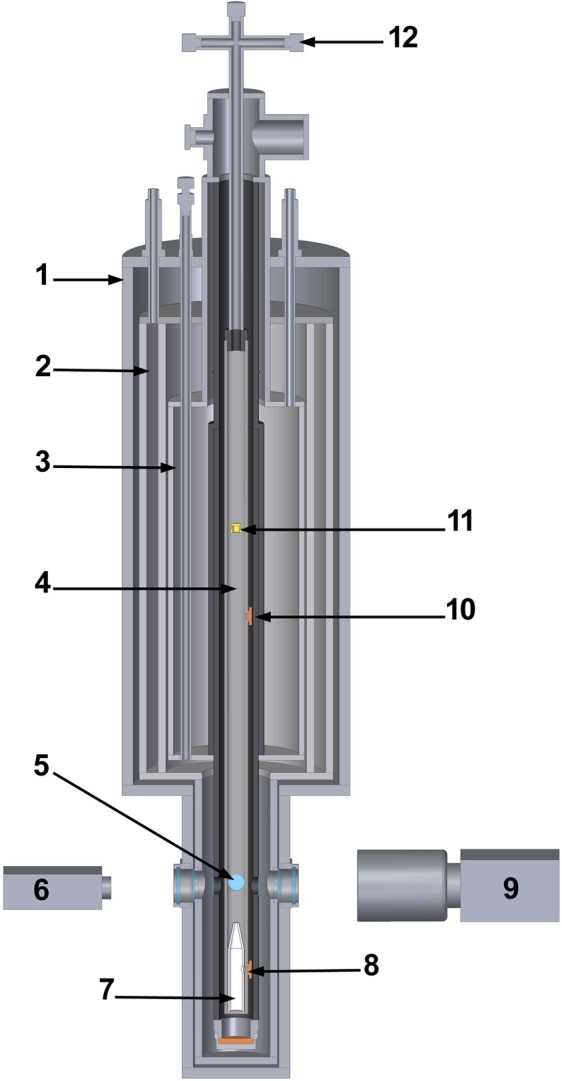
Scheme of the experimental setup: (1) cryostat; (2) liquid nitrogen bath; (3) liquid helium bath; (4) gas discharge tube; (5) dusty plasma structure; (6) laser; (7) dielectric cone; (8) cathode; (9) high-speed video camera; (10) anode; (11) injector of CeO_2_ particles; (12) cross-shaped connector.

The experimental setup for investigation of cryogenic helium plasma and dusty plasma structures has been described in more detail elsewhere^32^. The setup to study dusty plasma structures at liquid helium temperatures was developed based on a Janis SVT-200 optical cryostat. A scheme of the setup is shown in Fig. 1. The temperature of liquid helium in the cryostat could be lowered down to 1.6 K by pumping helium gas away. Experiments with DC discharge were carried out in a vertically oriented glass tube (4) placed in the inner channel of the cryostat. The lower end of the discharge tube (up to the position of dusty plasma structure) was emerged into liquid helium at temperatures below 4.2 K. The inner diameter of the tube was 20 mm and the distance between electrodes was 600 mm. The pressure of helium gas in the discharge tube was measured with a Granville-Phillips 275 convectron attached to a cross-shaped connector (12). Two thermometers were used for temperature measurements in the cryostat. The former was located close to the lower end of the discharge tube. The latter was fixed on the outer surface of the discharge tube at the height of dusty plasma structure (5).

At the beginning of the experimental procedure the temperature in the inner channel of the cryostat was lowered down to 1.6 K, then the channel was filled with superfluid helium (HeII) up to its optical windows. DC discharge inside the discharge tube cooled with HeII was initiated by stressing cathode (8) with negative potential of −3.2 kV. The helium gas pressure of 4–6 Pa was kept in the discharge tube. The discharge current was about 20–30 μA. Such glow discharge regime, with current less than 100 μA and no light emission from the discharge region, is optimal for keeping the helium gas temperature close to the temperature of the tube wall 4.2–5 K^21^.

Dusty particles were introduced into the discharge region by injector (11) located in the upper section of the discharge tube. The injector was filled with polydisperse CeO_2_ particles (with size from 0.1 μm to 100–200 μm). These injected particles fell into the positive column of the discharge, where their charging and trapping in ionization regions (strata) occurred. Thus, dusty plasma structures are formed. The dusty plasma structures formed in the lowest stratum of the positive column were monitored and studied. The stratum position (5) was stabilized at the level of optical windows by inserting a special dielectric cone (7) which focused electronic flux into the tube axis. Motions of dusty particles in the plasma structure were monitored by high-speed video camera (9) with a rate up to 300 frames/s. Dusty plasma structures were illuminated with a laser “knife” (with a height of 8 mm and a width of 0.22 mm) introduced into the cryostat through an optical window at the right angle to the axis of the high-speed video camera. A continuous wave solid-state laser (6) providing up to 85 mW at 532 nm was used for illumination.

## Results and Discussion

**Figure 2:**
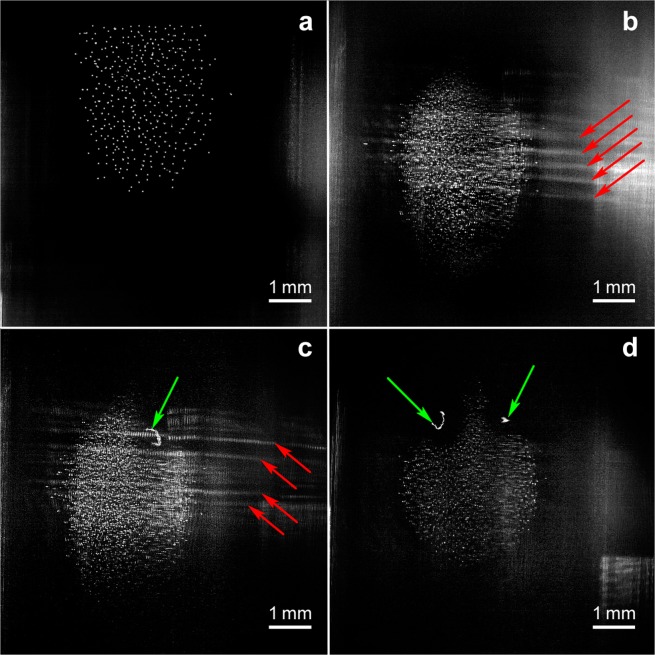
Dusty plasma structure transformation within the temperature range from 1.63 up to 2.16 K: (**а**) dusty plasma structure formed with CeO_2_ particles at 1.63 К; (**b**) dusty plasma structure and waves in a cloud of polymer nanoparticles, Т = 2.0 K; (**c**) dusty plasma consisting of CeO_2_ particles, a cloud of polymer nanoparticles, and solid filament, Т = 2.0 K; (**d**) voids around solid filaments levitating inside the dusty plasma structure formed with CeO_2_ particles, Т = 2.16 K. Red arrows point to the density waves within the nanoparticle cloud and green arrows point to the solid filaments.

Transformation of the dusty plasma structure within the temperature range from 1.63 up to 2.16 K is shown in Fig. 2. The spheroidal dusty plasma structure at HeII temperature of 1.63 K is shown in Fig. 2a. The structure varied in its size from 2 to 5 mm, typically its height was larger than its diameter. The liquid-like structure consisted of chaotically moving fast and slow particles with their velocities varying by more than an order of magnitude. Some fast particles produced intense vortex flows on the lateral surface of the dusty plasma structure. The average interparticle distance in the structure, *l*_*ip*_, was 120 ± 15 μm.

The nanoparticle cloud appeared in the field of view approximately 1000 s after ignition of the glow discharge (Fig. 2b). The cloud height was similar to the height of the dusty structure, but its diameter was 3-fold greater than the diameter of the dusty plasma structure. The cloud was recognized due to the laser light scattering on its density modulations (the most intense density waves are marked with red arrows in Figs 2b,c) corresponding to the collective oscillating motion of the nanoparticles. It was found that the oscillation frequency decreased from 48 Hz down to 20 Hz when the temperature increased from 1.69 K up to 2.0 K at the pressure of ≈4 Pa. At the same time, the wave velocity decreased from 16.8 to 7.4 mm/s, while the wave length remained unchanged and was 0.37 ± 0.03 mm. No voids were observed around the dusty particles within the superimposition area of the dusty structure and the nanoparticle cloud.

Separate solid filaments started to enter and leave the field of view a few minutes after the appearance of the cloud. The filaments are marked with green arrows in Figs 2c,d. Short filaments with the length of ~0.1 mm spun rapidly. Their rotational speed exceeded 100 turns/s.

No voids were observed around the filaments in the nanoparticle clouds (Fig. 2c), however there were voids around the filaments in the dusty plasma structure. The gaps around the filaments in the dusty structure were substantial, 0.3–0.4 mm in size (see Fig. 2d). The filaments usually levitated at the same height relatively to the spheroidal structure center. The nanoparticle cloud was observed within the temperature range of 1.6–2 K. The filaments were visible in the field of view up to the temperature of 4.4 K.

Upon collapse of the dusty plasma structure at 9.8 K because of turning off a discharge the microscopic particles were thrown by decaying electric field on the tube wall. The discharge tube was removed from the cryostat after each experiment. The CeO_2_ particles stuck to the tube wall and formed the “print” of the dusty structure on the inner surface of the tube. The particles and filaments settled on the tube wall were collected with a carbon tape and investigated by scanning electron microscopy and x-ray energy dispersive (EDX) microanalysis.

It was found that the cerium oxide particles collected from the tube wall have a narrower size distribution (from 1 to 20–30 μm) compared with the initial broad distribution of the polydisperse particles (from 0.1 to ~100 μm). Effective charge value, *Z*_*d*_, for a single particle with 1 μm diameter, has been obtained using the balance of the gravitational and electrostatic forces (assuming that the cerium oxide density is 7.6 g/cm^3^ and vertical electric field gradient is 10 V/cm) and found to be ≈250*e*, where *e* is elementary charge value, 1.6 × 10^−19^ C. Based on this charge value, the coupling parameter, *Г*, has been estimated. *Г* reflects the ratio between the potential (Coulomb, *E*_*C*_) and thermal (kinetic, *E*_*k*_) energies of the particles. The coupling parameter value is determined by using the formula:

$$\Gamma ={E}_{C}/{E}_{k}=1/(4{\rm{\pi }}{\varepsilon }_{0})\times {({Z}_{d}\times e)}^{2}/({E}_{k}\times {l}_{ip})$$

where ε_0_ is the dielectric constant.

A value *Г* ≈ 20 has been obtained for *E*_*k*_ = *m*_*p*_ × < *v*_*p*_ > ^2^/2 ≈ 6 × 10^−21^ J determined from the values of the mass of a CeO_2_ particle with 1 μm diameter, *m*_*p*_, and the average velocity, *v*_*p*_, of the dusty particles over the structure, obtained from video records processing. This value is in a good agreement with the liquid-like spheroidal dusty plasma structure observed. Thus, a rough estimation of densities of electrons and positive ions in the quasi-neutral complex plasma can be made, using concentration of the dusty particles, *n*_*d*_ = 5 × 10^5^ cm^−3^, calculated from the average interparticle distance, *n*_*e*_, *n*_*i*_ ~* n*_*d*_ × *Z*_*d*_ ≈ 10^8^ cm^−3^.

**Figure 3:**
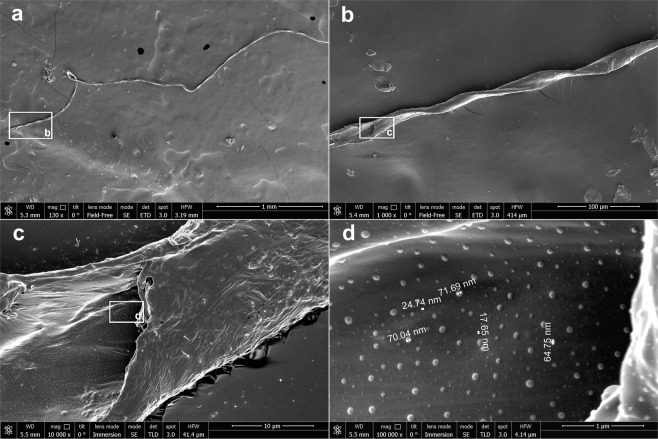
SEM micrographs of a helical filament obtained at different magnifications: (**a**) х130; (**b**) х1000; (**c**) х10000; (**d**) х100000. White frames correspond to the area shown in next micrographs.

The filaments are elastic helical bands with the widths from 12 to 22 μm and the thickness 2–5 μm. Electron micrographs of a filament with the length of 5 mm are given in Fig. 3. Micrographs with different optical magnifications are shown to provide more information on the filament structure. White frames in Figs 3a–c refer to the area shown in Figs 3b–d, respectively. The general view of the filament and its end are shown in Fig. 3b, respectively. The specific mass of the filament has been estimated and found to be 0.2 μg/mm assuming that the specific weight of the filament is about of 1 g/cm^3^. Thus, the specific charge required for the filament levitation should be as high as ≈7 × 10^6^ *e*/mm. The helical band looks like an Archimedes’ screw. Small convexities can be seen on the filament surface at higher optical magnifications, Figs 3c,d. Their diameters are within the range from 10 to 100 nm. We suggest that these convexities correspond to nanoparticles in the cloud observed at temperatures below 2 K. Small sizes of these nanoparticles and the absence of voids around the filaments levitating in the nanoparticle cloud point together to low values of their electric charge, ~*e*.

The chemical composition of the filaments was studied by x-ray energy dispersive microanalysis. The filament composition is given in the Table 1 and compared with the composition of the dielectric cone made of DAS clay (modeling material, air-hardening). The artificial clay consists of talc, Mg_3_(OH)_2_Si_4_O_10_, ≈30 wt%, gypsum, CaSO_4_x2(H_2_O), ≈30 wt%, and cellulose, (−C_6_H_10_O_5_−), fibers, ≈30 wt%^33^. The remaining component of the clay is an acrylic glue^34^ with the composition covered by a patent^33^. The data shown in the Table 1 reveal significant difference in compositions of the filaments and the clay. It can be seen that the filaments consist mainly of carbon, oxygen, and silica. An appearance of cerium in the filaments may be explained by adsorption the cerium oxide particles on the filament surface. The chemical composition of nanoclusters was not investigated because of their small size (Fig. 3d).

**Table 1:** Chemical compositions of the clay and the filaments (EDX microanalysis did not detect hydrogen atoms).

Clay		Filament	
Chem. element	Atomic weight, %	Chem. element	Atomic weight, %
O	**61**	**O**	**30**
C	**14**	**C**	**61**
Mg *	**11**		
Si	**9**	**Si**	**8**
Al *	**3**		
S *	**1**		
Ca *	**1**		
		Ce**	**1**

*The contents of Mg, Al, S, and Ca in the filaments were of ~0.1%.

**A presence of cerium in the filaments may be explained by adsorption the cerium oxide particles on the filament surface. Some other chemical elements (Na, K, Cl) were detected in the clay and in the filaments as impurities at the levels ~0.1%.

It can be concluded that intense sputtering of the dielectric cone used for stabilization of the lowest stratum position in the positive column of the glow discharge occurs due to electrons and ions focused into a small hole at the top of the insert. It is known that sputtering of a solid polymer target at room temperature may lead to a release of volatile fragments of macromolecules which can be further used as precursors for plasma polymerization^35–37^. In many cases, sputtering of polymers forms structures which chemically resemble neither the virgin polymer nor the polymer prepared by plasma polymerization of the monomer because any mass transported is likely to be highly reactive atoms and small polymer fragments, and not long intact chains^36^. Moreover, different polymer fragments may form different structures. For example, radio frequency magnetron sputtering of poly(tetrafluoroethylene) produced super-hydrophobic films in which cross-linked nanoparticles of fluorocarbon plasma polymer were embedded in an uncross-linked continuous (-CF_2_-)_x_ matrix with highly crystalline structure^37^. The 20–30 nm nanoparticles formed in proximity of the magnetron and had cross-linked structure because of fast random radical recombination in the plasma. The continuous phase was formed at remote distances from the magnetron as a result of slower step-growth gas phase polymerization of CF_2_ bi-radicals.

It is worth noting the steady state conditions of discharge at liquid helium temperatures: the pressure in the discharge tube depended only on the temperature of liquid helium and the discharge current. The sputtering of the dielectric insert at room temperature causes a fast pressure growth in the discharge tube and results in a bright emission from the area close to the cone hole. The color of the emission from this area was changing in time. The final pressure in the discharge tube, after turning off the discharge and cooling down the helium gas, could be 2–3 fold higher than the initial pressure (~100 Pa). This pressure increase can be related to efficient sputtering of the dielectric insert. Some of sputtered material remains in the gas phase at room temperature providing additional pressure in the tube and changing the emission spectrum. It is evidently that any sputtered material will aggregate immediately into nanoclusters either precipitate on cold surfaces at temperatures of liquid helium (vapors of any substance, excluding helium, are supersaturated at T = 2 K, even the pressure of molecular hydrogen at such temperature is far below than 10^−5^ Pa).

We also suggest that the temperature of neutral gas inside the discharge tube is approximately the same as the temperature of the tube wall. This suggestion is based on the following experimental facts: very low heat release, ~1 mW/cm, was in the discharge region filled with rather dense (≈2 × 10^17^ atoms/cm^3^) helium gas; no radial temperature gradient was observed through a void formation or rarefying the central part of the dusty plasma structure (Fig. 2). It is well known, that a radial temperature gradient (due to either fast cooling of the discharge tube wall or fast temperature increasing along the tube axes upon a current increase) makes dusty particles to move towards the tube wall due to the thermoforethic force^38^.

## Conclusions

A multimodal complex plasma formed by a spheroidal dusty structure consisting of polydisperse cerium oxide particles superimposed with a cloud of nanoparticles (less than 100 nm) and solid helical filaments (with the lengths up to 5 mm and the widths up to 22 μm) within the temperature range 1.6–2 K has been observed and studied for the first time.

The charges determined for nanoparticles, microscopic CeO_2_ particles, and solid filaments are ~1*e*, ≈250*e*, and ~10^6^*e*, respectively, and they are in good agreement with observations of cerium oxide particles interaction with nanoparticles and filaments.

The coupling parameter *Г*~10 was determined for the spheroidal dusty plasma structure and this value matches closely its liquid-like type.

The results obtained in the present work lead to a conclusion that the nanoclusters and solid filaments can be formed due to ion sputtering of dielectric material during the experiments at T < 2 K.

## Supplementary information

supplementary video
